# Evaluating the sensitivity of Halcyon’s automatic transit image acquisition for treatment error detection: A phantom study using static IMRT

**DOI:** 10.1002/acm2.12749

**Published:** 2019-10-06

**Authors:** Xenia Ray, Casey Bojechko, Kevin L. Moore

**Affiliations:** ^1^ Department of Radiation Medicine and Applied Sciences UCSD Moores Cancer Center La Jolla CA USA

**Keywords:** exit dosimetry, EPID, patient‐specific QA, transit images

## Abstract

**Purpose:**

The Varian Halcyon™ electronic portal imaging detector is always in‐line with the beam and automatically acquires transit images for every patient with full‐field coverage. These images could be used for “every patient, every monitor unit” quality assurance (QA) and eventually adaptive radiotherapy. This study evaluated the imager’s sensitivity to potential clinical errors and day‐to‐day variations from clinical exit images.

**Methods:**

Open and modulated fields were delivered for each potential error. To evaluate output changes, monitor units were scaled by 2%–10% and delivered to solid water slabs and a homogeneous CIRS phantom. To mimic weight changes, 0.5–5.0 cm of buildup was added to the solid water. To evaluate positioning changes, a homogeneous and heterogeneous CIRS phantom were shifted 2–10 cm and 0.2–1.5 cm, respectively. For each test, mean relative differences (MRDs) and standard deviations in the pixel‐difference histograms (*σ_RD_*) between test and baseline images were calculated. Lateral shift magnitudes were calculated using cross‐correlation and edge‐detection filtration. To assess patient variations, MRD and *σ_RD_* were calculated from six prostate patients’ daily exit images and compared between fractions with and without gas present.

**Results:**

MRDs responded linearly to output and buildup changes with a standard deviation of 0.3%, implying a 1% output change and 0.2 cm changes in buildup could be detected with 2.5σ confidence. Shifting the homogenous phantom laterally resulted in detectable MRD and *σ_RD_* changes, and the cross‐correlation function calculated the shift to within 0.5 mm for the heterogeneous phantom. MRD and *σ_RD_* values were significantly associated with the presence of gas for five of the six patients.

**Conclusions:**

Rapid analyses of automatically acquired Halcyon™ exit images could detect mid‐treatment changes with high sensitivity, though appropriate thresholds will need to be set. This study presents the first steps toward developing effortless image evaluation for all aspects of every patient’s treatment.

## 1 INTRODUCTION

As radiotherapy treatment plans have increased in complexity, the need for careful patient‐specific quality assurance (QA) has increased. Current recommendations for patient‐specific QA are focused on pretreatment verification^1^, ^2^, ^3^, ^4^ where the patient’s plan is delivered to either a phantom or air and the absolute dose is measured using ion chambers, film, or the electronic portal imaging device (EPID).^5^ These techniques can be time‐consuming and will not detect errors due to improper patient setup, changes in the patient’s weight, or corruption of the treatment plan file that occur after the creation of the verification plan. As a result, various groups have investigated the potential of using the EPID to measure patient exit dosimetry in hopes of catching these failure modes.^6^, ^7^, ^8^, ^9^, ^10^, ^11^ In exit dosimetry, EPID is positioned behind the patient during treatment and detects the full exit fluence from each beam. The panel is calibrated^7^, ^9^, ^12^, ^13^, ^14^, ^15^, ^16^ to convert the results to dose. The EPID‐measured dose can then be compared to a precalculated expected dose^10^ or back‐projected^17^ to determine the 3D dose that was deposited in the patient. With both techniques, the EPID monitors the entire treatment process and thus has the potential to detect errors in the beam delivery (MLC motion, beam output) and changes in the patient (motion during treatment, weight changes). While back‐projection gives more information about the delivered dose distribution in the patient, comparing the EPID measured dose to a forward calculated prediction can be faster and would allow for detecting differences mid‐treatment in time to correct them.

While several research groups have developed highly accurate techniques to acquire and analyze EPID transit images,^10^, ^11^, ^17^, ^18^, ^19^ few clinics are using exit dosimetry as part of their standard quality assurance.^20^ This slow adoption can be partially attributed to resistance to changes in the clinical work flow, an increased risk of collision when the EPID is extended, and a lack of implementation guidelines. Consistent use of exit dosimetry for every fraction of every patient’s treatment would increase the frequency at which errors could be detected^21^ such as mid‐treatment motion or changes in the beam and thus increase patient safety and confidence in treatment delivery. This was demonstrated by the Netherlands Cancer Institute which used *in vivo* EPID dosimetry to analyze 4337 patient plans over four years.^20^ They detected 17 serious errors requiring intervention, nine of which would not have been detected with routine pretreatment quality assurance.^20^ They now use off‐line *in vivo* EPID dosimetry as part of their routine quality assurance and are evaluating modifications to perform online EPID dosimetry.^11^

Halcyon™ (Varian, Palo Alto, CA) is a new therapeutic linear accelerator available from Varian. By design, the EPID for this linear accelerator is always in the path of the beam, and this EPID automatically acquires portal images for all fields during clinical mode. Thus, exit dosimetry images are immediately available for analysis from every field of every patient’s treatment without any changes to the workflow. Additionally, as Halcyon™ is an enclosed gantry, there is no risk of patient or couch collision with the EPID.

The purpose of this study was to evaluate the sensitivity of the Halcyon™ imaging panel to changes in patient size, patient position, and beam output. Having an automated quantifiable way to identify changes in patient size is important as source to surface distance (SSD) values to the patient body cannot be measured on the Halcyon because it does not have an optical distance indicator (ODI). The KV‐CBCT which can be used to verify the body contour has not changed substantially but may not show the full extent for large patients. Additionally, kV imaging was not initially available on the Halcyon and the Halcyon can still be installed with only MV imaging. The MV imager has a smaller field of view so for all pelvic patients the body contour could not be checked for patient weight changes. The poorer soft tissue contrast with MV imaging could also lead to improper patient setup and thus having an independent patient positioning check after image guidance would be useful. Changes in patient position that occur during treatment after the initial image‐guided patient alignment are also important as they can have an adverse effect on the planned dosimetry particularly when tight margins are used or sensitive organs at risk are directly next to the target. On the Halcyon, we cannot use surface guided imaging to monitor intrafraction patient motion during treatment because of the bore, so using exit images would decrease the uncertainty in patient positioning. Additionally, errors in couch translations from image guidance could be detected with this method. Changes in beam output larger than those identified during daily output checks are exceedingly rare but would have a disastrous effect on patients. Failures in MLC trajectories will manifest from the EPID’s perspective as changes in beam output. Thus, adding an automated check for beam output will add an extra level of safety to our current clinical practice without requiring any changes to current workflow because these images are already being acquired.

This analysis provides a first look at whether this new device can easily and reproducibly detect changes in a patient’s setup or treatment to prevent an ineffective and potentially dangerous treatment from occurring. The eventual goal is to provide a quantitative metric that can be automatically measured from these images and compared to action‐level thresholds to impact clinical workflow. This would allow for immediate online verification that supplements pretreatment QA by effortlessly identifying errors that occur during treatment delivery including errors that would have previously remained undetectable.

## 2 MATERIALS AND METHODS

**Figure 1:**
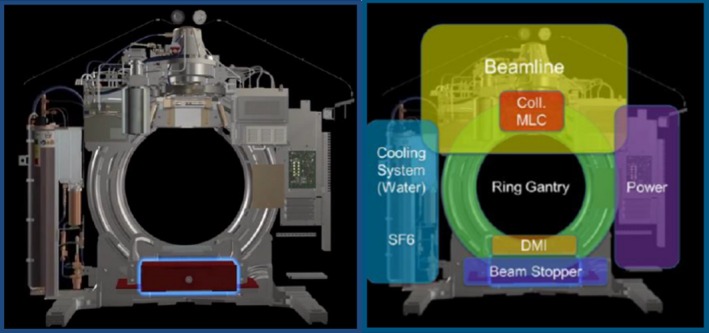
Diagram of the major internal components of the Halcyon™ linear accelerator.^31^ Note the Digital Megavoltage Imager (DMI) or electronic portal imaging device is mounted atop the beam stopper, rotates with the gantry, and is always in the beam path.

The Halcyon comes equipped with a Varian aS1200^22^, ^23^ digital megavoltage imaging panel (Varian Medical Systems, Palo Alto, CA) that is mounted directly opposite the single energy 6X‐FFF MV source, Fig. 1. The panel is located at a source to imager distance of 154 cm, has a physical size of 43 cm × 43 cm with a 28 cm × 28 cm isocentric projection, an image matrix of 1280 × 1280 pixels, and a projected pixel size of 0.22 mm in the isocentric plane. Images are acquired with 16 bit depth and a frame rate of 25 frames/sec. To prevent saturation during image acquisition, pixel “scaling is applied automatically if the intensity is close to the limit of the 16‐bit resolution.”^24^ This scaling factor is recorded in the DICOM header. To allow for equivalent image comparison, all processed image intensity values in this work were multiplied by this scaling factor prior to measuring the relative differences between images.

When operated in portal dosimetry mode, the panel integrates the readout obtained from the entire treatment field. This mode is most commonly used for patient‐specific intensity‐modulated radiation therapy (IMRT) QA to compare the predicted dose map to the acquired portal dosimetry image via gamma analysis.^25^ During treatment, this mode is automatically initiated for every field in a patient’s plan. The resulting exit dosimetry images are automatically exported to the record‐and‐verify system (ARIA, Varian Medical Systems, Palo Alto, CA) with no manual intervention required. Currently no vendor‐provided workflow is available for evaluating these images to detect changes in the patient setup and we do not currently have access to third party workflows.^10^, ^26^, ^27^, ^28^ Additionally, the treatment planning system (Eclipse, Varian Medical Systems, Palo Alto, CA) cannot currently generate a prediction exit image for comparison with these daily exit images as it does for portal dosimetry although some independent research groups have developed tools to accomplish this.^29^, ^30^

**Figure 2:**
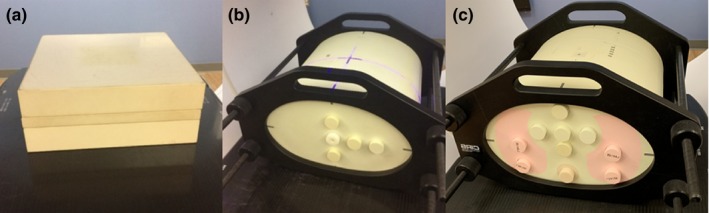
Images of the phantoms used in this study, (a) 12 cm stack of solid water, (b) CIRS intensity‐modulated radiation therapy (IMRT) homogenous phantom, and (c) CIRS IMRT heterogeneous phantom.

In this study, three types of clinical situations were simulated using phantoms: (i) changes in beam output, (ii) changes in patient weight, and (iii) changes in patient positioning. An open beam and a modulated beam were delivered for each test, both at a gantry and collimator angle of 0°. The open beam was a 10 cm × 10 cm square field with 100 monitor units (MUs). The modulated beam was from a prostate patient’s nine‐field IMRT plan and delivered 141 MU with an approximate field size of 8 cm^2^ × 8 cm^2^. Three phantoms were used for this study: a 12 cm high stack of solid water with width and length of 30 cm, a CIRS IMRT homogenous phantom (Model 002H5, CIRS Inc., Norfolk, VA), and a CIRS IMRT heterogeneous phantom (Model 002LFC) (see Fig. 2). All phantoms were set up with their midpoint at 100 cm SAD. After each test, the exit dosimetry images were exported from ARIA to MATLAB (The MathWorks, Inc. Natick, MA) for analysis.

### 2.1 Quantitative image analysis

The relative difference for each pixel between the baseline image and the subsequent test case was calculated for each detector pixel:

(1) $$RD_{\mathrm{ij}}=\frac{I_{0,ij}-I_{X,ij}}{I_{0,ij}}\times 100,$$

where *i* and *j* are the discretized positions in the X and Y directions; *I*_0,_ *_ij_* is the default image for a particular test (e.g. the exit dosimetry image from the solid water slab with no additional buildup); and *I_X,ij_* is the image being compared to the default image (e.g. the exit dosimetry image from the solid water slab with 1 cm of additional buildup).

Then the mean of these relative differences (MRDs) was calculated with the following equation,

(2) $$MRD\left(\%\right)=\frac{1}{N_{x}N_{y}}\sum_{\mathrm{ij}}^{N_{x},N_{y}}RD_{\mathrm{ij}},$$

where *N_x_* and *N_y_* are the total number of pixels in the image in the X and Y directions, respectively.

The standard deviation of the relative differences between each pair of pixels was also calculated to establish the uncertainty in each measurement:

(3) $$\sigma_{\mathrm{RD}}=\sqrt{\frac{\sum_{\mathrm{ij}}^{N_{x}N_{y}}{\left(RD_{\mathrm{ij}}-MRD\right)}^{2}}{N_{x}N_{y}-1}}$$

To determine if the measured MRD would be detectable, 10 repeat images of both the modulated and open fields were acquired with the CIRS homogeneous phantom with no change in the acquisition conditions. The average of these 10 images was calculated and used as the baseline image. Then the MRDs between the 10 images and the baseline image were calculated. The standard deviation in these 10 values for the MRD was calculated with,

(4) $$SD_{\mathrm{MRD}}=\sqrt{\frac{\sum_{k=1}^{N_{\mathrm{meas}}}{\left(MRD_{k}-\bar{\mathrm{MRD}}\right)}^{2}}{N_{\mathrm{meas}}-1}},$$

where *N_meas_* is the number of repeat images. *SD_MRD_* estimates the shot‐to‐shot noise in the system. Thus, measured differences between images under different setup conditions must result in values greater than 2.5 times the *SD_MRD_* in order for the change to be detected with 2.5‐sigma confidence.

To define the usable “in field” component of a modulated beam, we set a minimum pixel intensity threshold of 50% of the mean image value in the baseline image. The same pixel locations that were excluded in the baseline image were also excluded from the compared images.

### 2.2 Output change detection

Linac output is checked every morning prior to patient treatment and is unlikely to change significantly during the day, however, failures of MLC trajectory would appear as relative changes in output and have been documented as clinical errors. To simulate an error in the output, an open beam and a modulated beam were delivered to each phantom six times with the following relative decreases in the monitor units: 0%, 2%, 4%, 6%, 8%, and 10%. For each deviation in monitor units, the MRD and *σ_RD_* were calculated by comparing each image with the scaled change in MUs to the default image with no change to the MUs. The response of the MRD was compared to the introduced scaling in the output. Additionally because this relationship was expected to be linear,^21^, ^32^ a least squares linear regression analysis was applied to each set of data points and the coefficient of determination was calculated.

### 2.3 Weight change detection

On our other treatment machines, weight changes are often noticed during weekly checks when the daily recorded SSD values are evaluated by physicists. Halcyon does not have an ODI so SSDs are not recorded, and many Halcyons are equipped with only MV imaging which leads to substantial truncation of the body contour. Thus a simple quantifiable metric from exit images could serve the same purpose and potentially detect changes earlier. To simulate a change in the patient’s weight, extra buildup was incrementally added to the 12 cm stack of solid water phantom. Extra solid water was added on top of this phantom in steps of 0.5, 1.0, 1.5, 2.0, 2.5, 3.0, 4.0, 5.0 cm resulting in total thicknesses ranging from 12 to 17 cm. For each thickness, both the open beam and modulated beam were delivered. The MRD and *σ_RD_* were calculated from the images acquired after each increase in buildup to the default image from the delivery with the baseline of 12 cm thickness. This test was only performed on the solid water phantom because of the inherent difficulty in adding symmetrical buildup on top of the curved CIRS phantoms. The resulting measured MRDs were evaluated to determine if the panel response was linear to the proportional changes in spectrum and attenuation. This relationship was also expected to be linear, so a least squares linear regression analysis was applied to each set of data points and the coefficient of determination was calculated.

### 2.4 Position change detection

**Figure 3:**
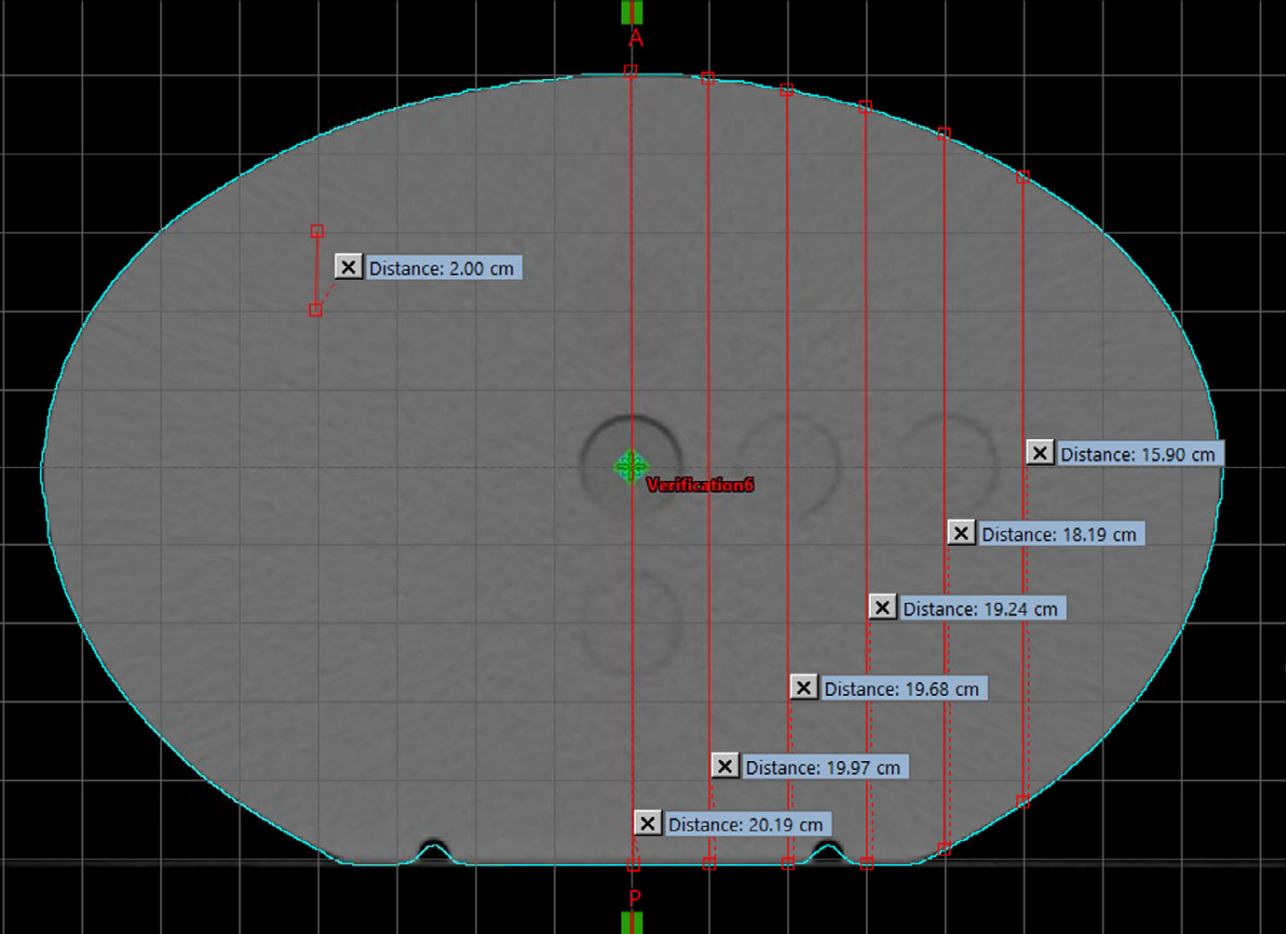
An axial computed tomography slice of the CIRS homogenous phantom with a 2 cm × 2 cm grid overlaid. The thickness of the phantom intersected by the central beam at each shifted position is shown in red and ranged between 20.19 and 15.90 cm.

On the Halcyon, all patients are aligned using image guidance. Exit images could be used to detect incorrect couch translations from this alignment or changes in patient positioning due to intrafraction motion. To simulate the panel’s ability to detect large changes in the patient’s position from large motions during treatment or incorrect couch translations after image guidance, the CIRS homogenous phantom was shifted laterally. The phantom was centered with the lasers and the modulated and open beams were delivered. Then the couch was shifted 2, 4, 6, 8, and 10 cm to the left and each beam was delivered. Then the couch was shifted 2, 4, 6, 8, 10 cm to the right from the baseline and the modulated beam was delivered. For each shift, the MRD and *σ_RD_* were calculated using the image acquired with the phantom centered as the baseline. The CIRS phantom is oval shaped and so the relative thickness that the beam is passing through changed with each shift, mimicking a patient’s inhomogeneous body habitus. An axial computed tomography (CT) image of the phantom was used to measure the thickness of the phantom intersected by the central beam axis at each shifted point, Fig. 3. Measurements using the open field were only measured with shifts to the right, because the beam and phantom are symmetric.

Effects of smaller more clinically likely patient shifts were investigated using the heterogeneous CIRS phantom. Shifts of 0.0, 0.2, 0.4, 0.6, 0.8, 1.0, and 1.5 cm were introduced. The MRD and *σ_RD_* were measured from the images acquired after each shift using the modulated field. Additionally, the magnitude of shift was calculated from the images acquired using the open 10 × 10 field. To calculate the shift, a MATLAB script was written which first applies an edge detecting Sobel filter^33^, ^34^ to the images and then converts each filtered two‐dimensional image matrix into a long, one‐dimensional array. A cross‐correlation^35^ function is then performed between the array from the baseline image and the array from the shifted image. From the results of the cross‐correlation function, the number of pixels required to shift the row from the second image so that it best aligns with the row from the upshifted image can be determined. Each pixel is 0.22 mm wide so this allows us to compare the calculated shift with the magnitude of the physical shift that was performed. As we knew 1D shifts had been applied, we used a 1D cross‐correlation function. In a clinical setting, where the shift is unknown, a 2D cross‐correlation function could be used. The edge detecting filter is used on the image first to highlight areas that can be used for alignment. Without an edge detecting filter, the cross‐correlation function can erroneously attempt to minimize differences in CT noise versus large differences at interfaces.

### 2.5 Patient study

To better understand the potential of this technology for a patient case, the daily exit dosimetry images for six prostate patients’ treatments who were observed to have gas during some of their treatments were also analyzed. We selected for this variable as it can be determined retrospectively and could have a harmful impact on their delivered versus planned dosimetry. These patients were treated at our institution using a nine‐field IMRT plan for 50 to 56 Gy in 25–28 fractions on the Halcyon™. The field at gantry 0 (anterior‐posterior incidence for head‐first‐supine positioning) from the first patient’s plan was used in the phantom studies described above. The exit dosimetry images acquired with the gantry at 0 for each of the patients’ treatments were exported from ARIA. Ideally, a predicted exit dosimetry image could be generated by the treatment planning system for comparison to the daily acquired images. However this feature is not currently available in our treatment planning system version (Eclipse v.15.1, Varian Medical Systems, Palo Alto, CA) so one of the daily images was used as the baseline in the calculations. We observed large gas bubbles within the treatment field on the first fraction for several of the 6 patients we selected. Rather than comparing all the images to an image with a large bubble, we selected the first fraction without a gas bubble for each patient as the baseline. In future prospective applications for on‐treatment monitoring, the first fraction’s image could be utilized to ensure consistency. For the purposes of this work, we sought to understand the day‐to‐day variations present in real cases. All of the images were categorized as having or not having gas. T‐tests were conducted to comparing the MRD and *σ_RD_* values for images with and without gas for each patient. Because of the number of tests we conducted, multiplicity correction using the Bonferroni^36^ technique was applied to these *P*‐values. Corrected *P*‐values < 0.05 were considered significant. In addition to measuring the MRD and *σ_RD_*, these images were also visually examined for anatomical features that could be used to detect mid‐treatment changes in the patient positioning or setup. Shifts in the pubic symphysis were measured manually using ImageJ (ImageJ 1.5k, NIH, Bethesda, MD).

## 3 RESULTS

### 3.1 Phantom studies

The repeatability test using the CIRS homogeneous phantom resulted in a measurement standard deviation of *SD_MRD_* = 0.14% for the open fields and *SD_MRD_* = 0.30% for the modulated fields. As Halcyon™ is currently configured to only treat with modulated fields, the latter value is likely a better representation of the practical detectability. The maximum values for the MRD for the 10 identical repeated images were 0.24% for the open field and 0.67% for the modulated field while the averages were effectively 0.

**Figure 4:**
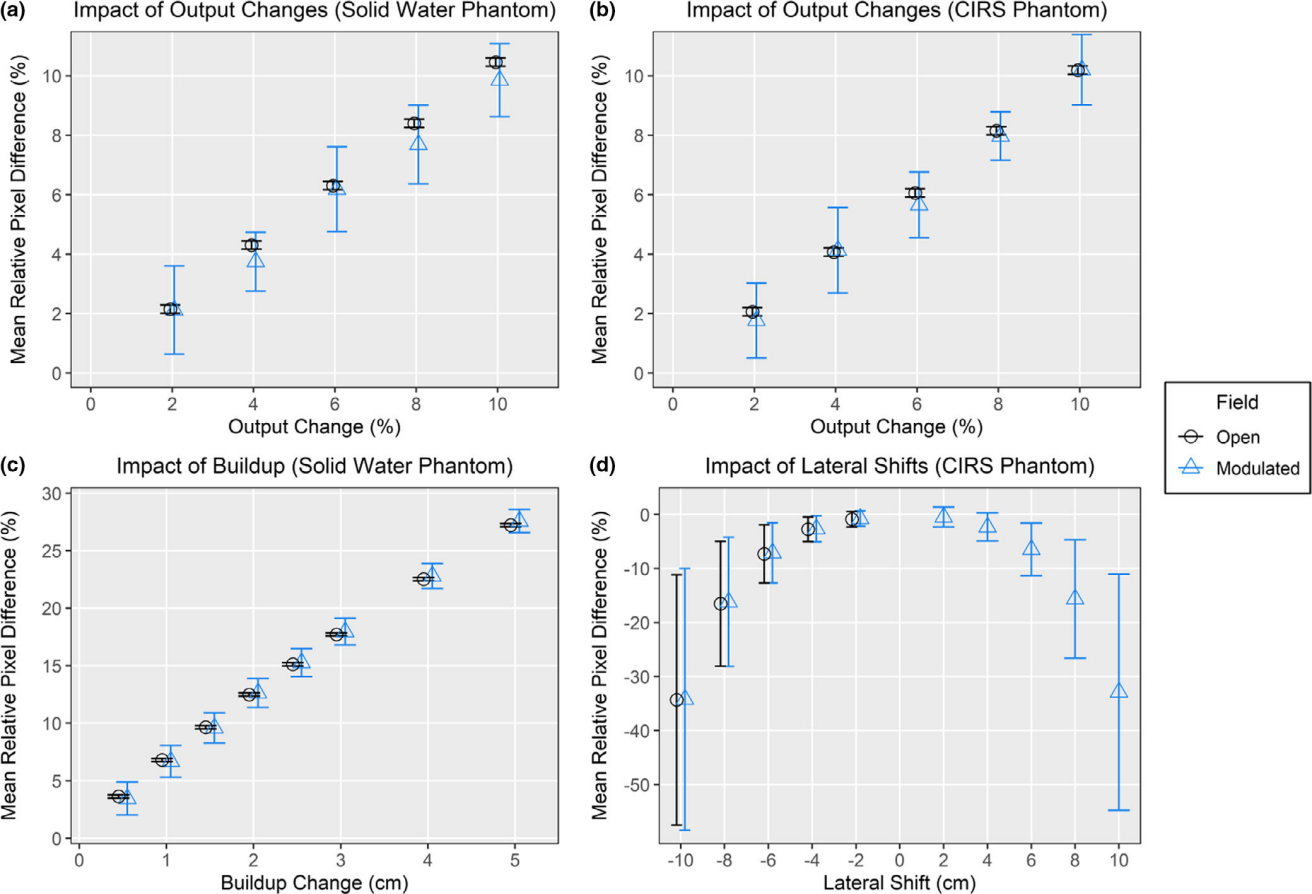
Results of the exit panel dosimetry analysis using a solid water and homogenous CIRS phantom for three tests: (a,b) decreasing the monitor units, (c) adding extra buildup material, and (d) performing lateral shifts. Each test was performed with an open 10 × 10 beam and a modulated beam. The standard deviations for the output and buildup plots are the values of *σ_RD_* or *SD_MRD_*, whichever was larger for that test.

The results of the three tests of the imager’s response using phantom data are seen in Fig. 4.

When the MUs were decreased by 2%–10% the MRDs for both plan types (open and modulated) and both phantom types (solid water and CIRS) were linearly proportional [Figs. 4(a) and 4(b)]. Linear regression lines through the data had slopes of 1.04 (r^2^ = 0.999), 0.97 (r^2^ = 0.999), 1.02 (r^2^ = 0.996), and 1.04 (r^2^ = 0.996) for the open field in solid water, modulated field in solid water, open field in the CIRS, and modulated field in the CIRS phantom, respectively. For a 2% decrease in the monitor units, which is the most clinically relevant scenario, the MRDs were between 1.8% and 2.1% for the four field and phantom combinations with the furthest off MRD value occurring for the modulated field after it traversed through the CIRS phantom. The errors between the MRD and the predicted differences were all less than 0.5% for both phantoms with the largest error of 0.46% occurring for the 10% increase in output with the open field through the solid water phantom. The *σ_RD_* values were within 1.5% for each of these image comparisons.

When extra buildup was added to the solid water phantom to simulate a change in the patient’s weight, the MRD also changed linearly. The calculated relative change per cm for these data [Fig. 4(c)] using a linear fit was 5.2% (r^2^ = 0.997) and 5.3% (r^2^ = 0.997) for the open and modulated fields respectively. This was 1.2% and 1.3% larger than the 4%/cm predicted change from the tissue phantom ratio (TPR) which had been measured and verified in our phantom stack using a Farmer chamber (PTW N23333, PTW‐Freiburg, Germany). However, the TPR is defined for the beam profile within the phantom and it is known that changes in the profile occur between when it exits the phantom and is incident on the flat panel imager (e.g. increased proportion of scatter).^29^, ^37^ Additionally, studies have shown that flat panel imagers are over responsive to lower energy photons.^29^, ^37^ Thus any beam hardening or increased scatter proportion can result in larger than expected relative differences measured by the imager.

To isolate the Halcyon™ detector response, we compared it to the response from a Varian TrueBeam™ linear accelerator at our clinic. Our TrueBeam uses the same model EPID (as1200) as the Halcyon™ but allows for placing the ion chamber behind the detector and changing the detector distance. Images were acquired with the 12 cm solid water stack with no extra buildup, and 1, 5, and 9 cm extra buildup. For each measurement, 200 MUs were delivered with a 10 cm × 10 cm open field with the imager at 154 SID. Then a Farmer chamber (PTW N23333, Germany) with buildup cap was placed first on the proximal and then distal side of the TrueBeam’s imager to measure the relative differences in collected charge for the four solid water thicknesses. The relative differences in the central pixel of the imager and ion chamber measurements for each setup were calculated. Results are presented in Table 1.

**Table 1:** Measured relative differences for the EPID and ion chamber with extra solid water buildup.

Extra buildup (cm)	Relative differences from baseline (no extra buildup)			
	Halcyon™ EPID (%)	TrueBeam EPID (%)	Ion chamber above the EPID (%)	Ion chamber behind the EPID (%)
1	6.30	6.30	5.09	4.87
5	26.95	26.69	22.90	22.28
9	41.81	41.35	36.86	36.18

EPID, electronic portal imaging device.

We observed that the EPID on the TrueBeam had the same response to extra attenuation material as the Halcyon™ imager with all values being within half a percent. The ion chamber measurements verified that the imager was under‐responding by approximately 1%/cm due to the slight beam hardening.

The *σ_RD_* values for the added build up images were all under 1.5% with a max value of 1.43% for 0.5 cm added buildup with the modulated field. *σ_RD_* for the open field ranged from 0.07% to 0.08% and exhibited no trend with increasing buildup. *σ_RD_* values for the modulated field were slightly larger and ranged from 1.01% to 1.43%. Additionally the *σ_RD_* values for the modulated field decreased linearly with increasing buildup.

In the third test, the homogeneous CIRS phantom was shifted to simulate patient positioning changes. The resulting MRDs followed a clear trend, Fig. 4(d). For the modulated and open fields, the MRD increased with the increased magnitude of the shift. For shifts greater than 2 cm, the modulated field had larger values for the MRD than the open field and this separation increased with increasing shift sizes. For the most clinically relevant shift of 2 cm, the open field had an MRD of only −0.90% and the modulated field also had small MRDs of −0.47% and −0.76% for shifts in the positive and negative direction, respectively. This corresponds to a thickness change along the central beam axis of only 0.21 cm. The values for *σ_RD_* increased substantially as the shifts increased as seen in Fig. 4(c). For the open field, *σ_RD_* ranged from 1.43% to 23.14% for the 2‐10 cm shifts, respectively, while for the modulated field *σ_RD_* ranged from 1.39 to 24.2. This suggests that while *σ_RD_* is a more sensitive metric than MRD for detecting lateral shifts, neither is particularly sensitive nor prescriptive of in what direction the patient needs to be shifted.

**Figure 5:**
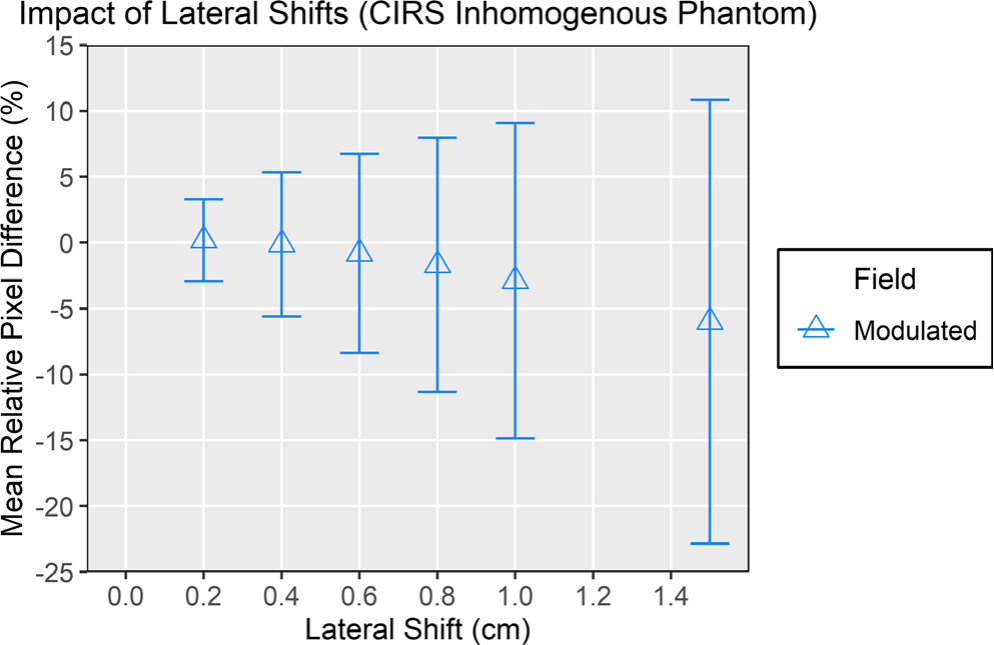
Results of the exit panel dosimetry analysis using a heterogeneous CIRS phantom to evaluate the impact of very small shifts on the mean relative difference and *σ_RD_*. The same modulated field was delivered at each shifted position. The standard deviations are the values of *σ_RD_* and increased substantially for these small shifts.

**Figure 6:**
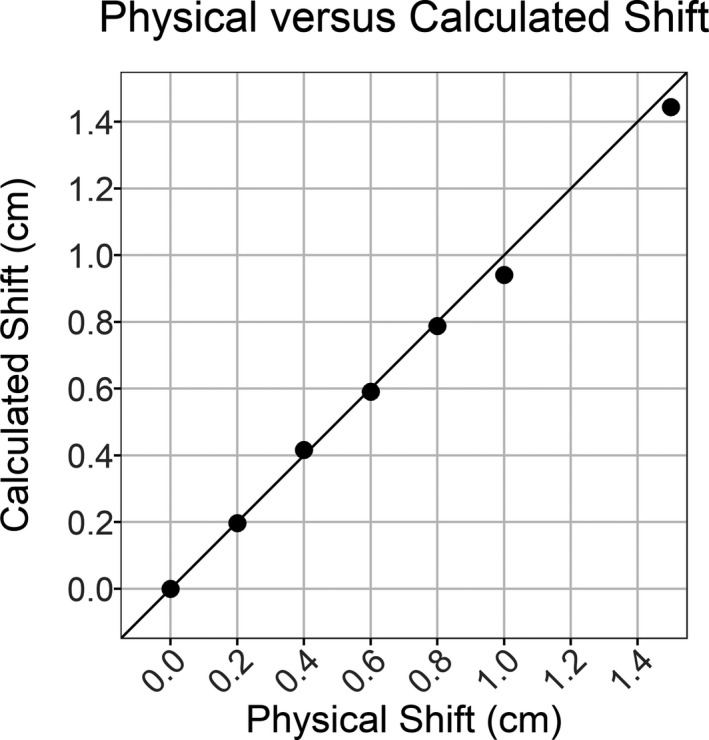
Values for the calculated versus actual shifts performed are plotted. For the six nonzero displacements, the average residual was 0.018 cm. A reference line with x‐y ratio of 1:1 is also plotted to help in highlighting the accuracy of the calculation.

The impact of lateral shifts was also assessed using the heterogeneous CIRS phantom and smaller shifts to demonstrate a more clinically likely scenario. Results are shown in Fig. 5, where for the smallest shift of 0.2 cm, the MRD was only 0.19% but *σ_RD_* was already 3.11%. This value for *σ_RD_* was larger than the corresponding values seen for up to 4 cm shifts in the homogenous phantom. As expected, both MRD and *σ_RD_* increased further with larger shifts but remained nonspecific to the direction or magnitude of displacement. The ability to calculate the magnitude of the introduced shift was investigated using cross‐correlation and the images from the open 10 × 10 field; results are shown in Fig. 6. The cross‐correlation was calculated between each image and the nonshifted image. For the six nonzero displacements, the average residual was 0.018 cm. The largest deviation in the calculated shift was at 1 cm where it was 0.059 cm less than the actual shift. Thus this metric was the only one with a linear response to increasing shifts and was the most accurate for analyzing the transit images.

### 3.2 Patient analysis

**Figure 7:**
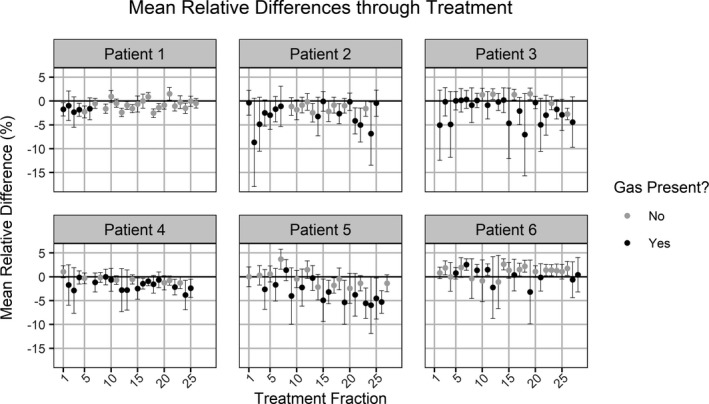
The mean relative differences between each treatment fraction and the patient’s baseline image. The standard deviations are *σ_RD_* values. Data values are shaded by whether the patient had gas at that treatment fraction.

**Figure 8:**
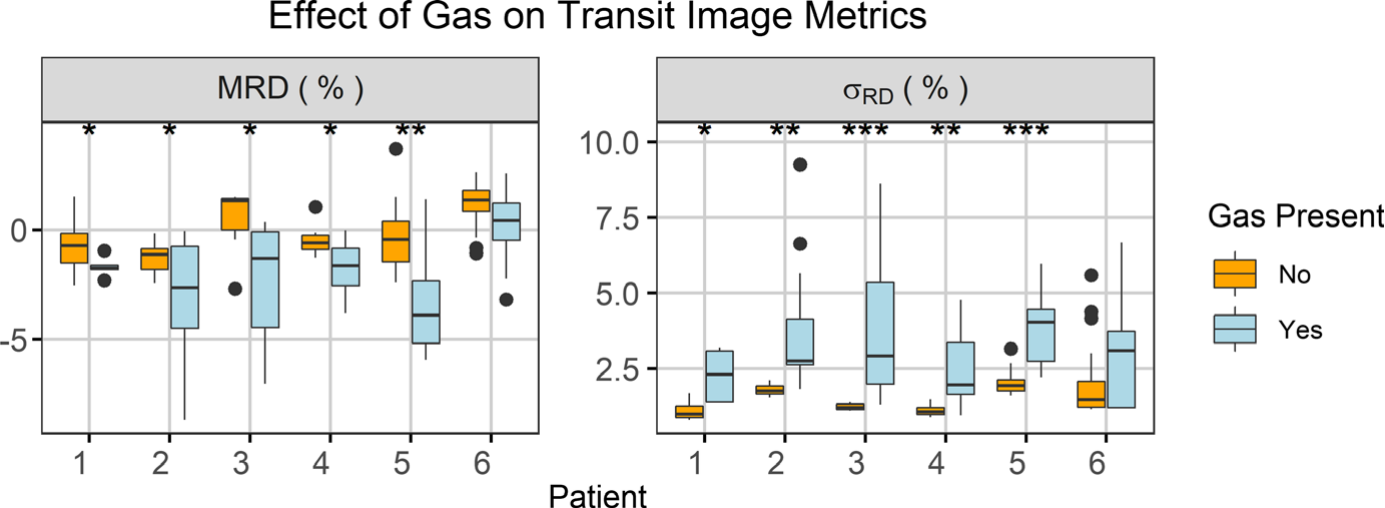
The effect of gas on the two metrics (mean relative difference and *σ_RD_*) was evaluated individual for each patient to determine if there was a significant difference. Five of the six patients had significant differences (corrected *P*‐value < 0.05) for both metrics. *P < 0.05, **P < 0.01, ***P < 0.001.

**Figure 9:**
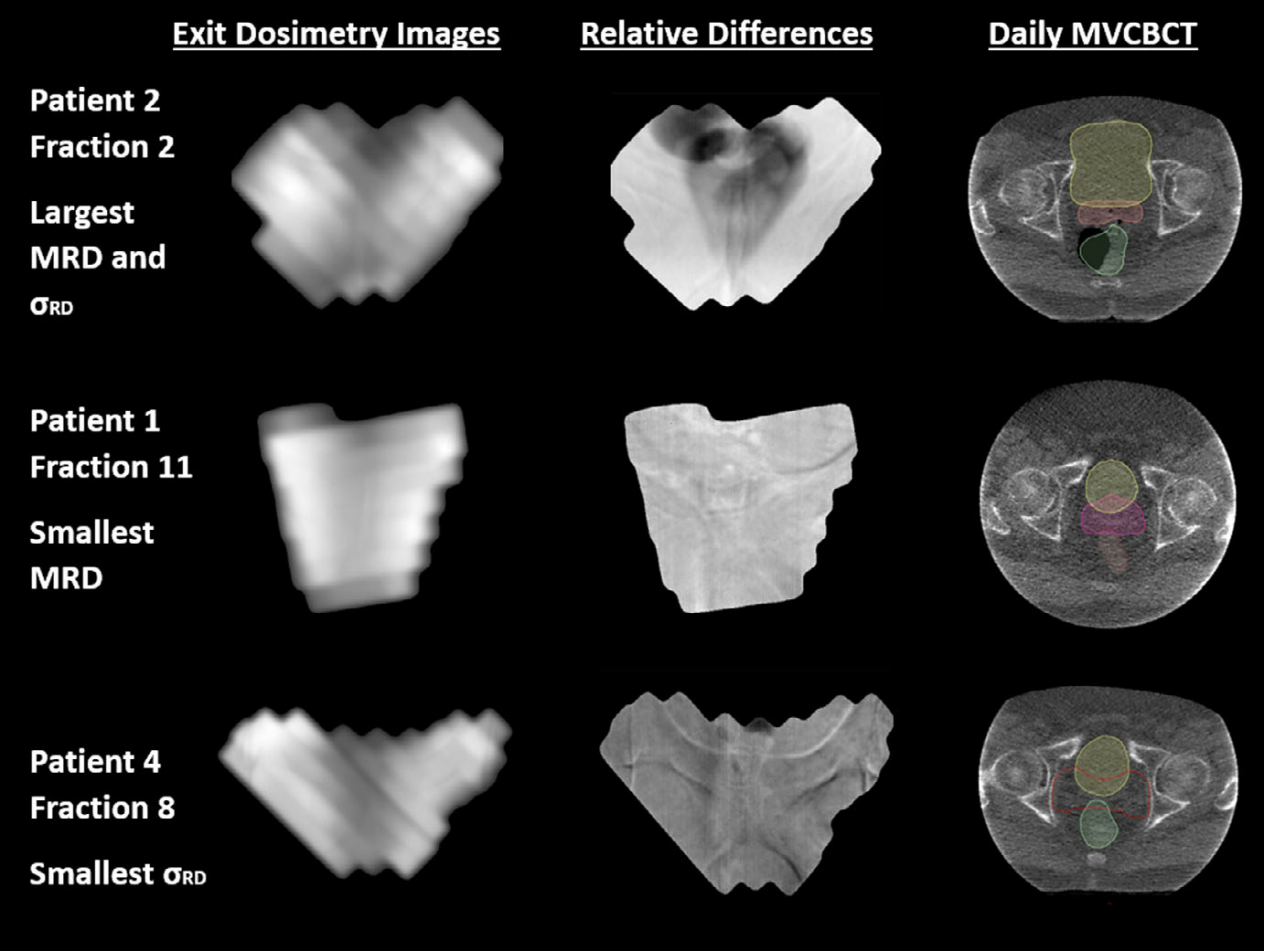
Examples of the patient exit dosimetry images from three fractions as well as the calculated relative difference between these fractions and the average image. A large gas bubble is immediately visible on the relative difference images from patient 2 fraction 2 which had the largest value for mean relative difference and *σ_RD_*. The pubic symphysis is apparent on all the calculated relative difference images. Note that the MV cone‐beam computed tomography images were acquired prior to setup correction.

Each patient’s daily exit images for one field were compared to their baseline images. Selected baseline images were from the first fraction where the patient did not have in‐field gas (fraction 1 for two patients, fraction 2 for one patient, fraction 6 for one patient, and fraction 8 for two patients). The presence of gas bubbles at individual treatment fractions was confirmed from the daily MV cone‐beam CT images the patients received. The results are shown in Fig. 7. Values for MRD and *σ_RD_* varied between patients and between fractions for an individual patient. Values for MRD ranged between −8.7% and 3.7% while *σ_RD_* values for ranged between 0.6% and 8.95%. Figure 8 shows the results of the t‐test comparing the values for MRD and with and without gas for an individual patient. Significant differences due to the presence of gas in the MRD and *σ_RD_* values were observed for five of the six patients. Without gas, the absolute value of the MRD values was below 3.7% while when gas was present, the absolute value of MRD values was all larger than 2.6% and up to 8.7%. Without gas, the *σ_RD_* values ranged between 0.5% and 4.1% but with gas included values between 0.8% and 8.9%. The MV cone‐beam CT (CBCT) and MRD images for those treatment fractions with the largest and smallest MRDs (patient 2/fraction 2 and patient 4/fraction 8) and largest and smallest *σ_RD_* (patient 2/fraction 2 and patient 1/fraction 11) were visually examined for noticeable features, see Fig. 9. The image with the largest value for the MRD and *σ_RD_* had a large gas bubble in the rectum that was visible on the MVCBCT and exit images. The pubic symphysis was visible in all the images and was observed to shift relative to its position on the first fraction by 1 to 2 mm in the 2D directions visible from the BEV with gantry at 0 when measured manually.

## 4 DISCUSSION

In this study, we set out to evaluate the sensitivity of the Halcyon™ imager to several potential on‐treatment changes. Our results indicated that the imager was sensitive to 2% changes in output, 0.2 cm lateral shifts, and 0.5 cm changes in buildup with MRDs between 1.6%–2.4%, 0.47%–0.76%, and 3.5%–4.0% for each of these tests, respectively. Additionally, the imager itself was very stable and had small values for *SD_MRD_* of 0.13% and 0.30% when repeated measurements were acquired of the open and modulated fields.

The changes in beam output resulted in a proportional change in the MRD. Thus even though we only tested changes of 2% or greater, it is likely that smaller changes could be detected. Additionally for this test, the resulting values matched the change in the output to within 1%. We tested large changes between 2% and 10% specifically to ensure that the panel had a linear response and that it would be capable of detecting catastrophic errors in a delivered plan (e.g. the MLCs not entering the field). When extra buildup was added or the patient was shifted laterally, which inherently changes the buildup, the results followed clear linear trends. For the added buildup, the MRDs were directly proportional to a slope of 5.2%/cm of extra buildup instead of 4%/cm as would have been expected from the TPR. However this variation in the behavior of the detector has been verified by other studies^29^, ^37^ which determined that the imager is oversensitive to lower energy photons, and when these are preferentially attenuated a proportionally larger reduction in the signal is observed. For this study and our intended eventual clinical implementation, it is important that the response was linear so that substantial patient weight changes would trigger large values for the MRD and signal the physicist to investigate further. Additionally, we verified our results for a subset of tests on a TrueBeam and thus accurate EPID estimates for the Halcyon™ would also apply to current C‐arm linacs.

For all three of the phantom tests, we measured both MRD and *σ_RD_* in the pixel pair differences. For the tests involving changes in beam output or buildup, the MRD was highly sensitive compared to *σ_RD_*. In these situations, the changes we introduced to the setup applied to the entire detector area and as such impacted each pixel pair in the same way. In contrast, when we shifted the homogenous CIRS phantom laterally in 2 cm increments, both the MRD and *σ_RD_* increased nonlinearly with the size of the shift. This can be attributed to the fact that the change in the amount of buildup is location dependent since the phantom is oval shaped and so the response at the edge of the field is changing much more than near the center. The calculated shift using the cross‐correlation function was a more sensitive metric for evaluating the magnitude of changes in position when tested with the open field and a heterogeneous phantom. By measuring these metrics from routine clinical images, we could potentially decouple changes in beam output from changes in position in order to determine sources of error. The calculated shift magnitude was highly accurate and thus is the best metric for evaluating shifts. Future analyses will include shifts in more than one direction and with rotations to better represent the variety of positioning changes that are experienced in patient data.

Quantitative thresholds for the different metrics will allow for treatments to be flagged for review by physicists similar to how currently couch position overrides or measured differences in SSDs over the course of treatment trigger physicists to conduct a more in‐depth review during their weekly check. It is worth noting that Halcyon does not have an optical distance indicator so SSDs cannot be recorded and on the daily imaging CBCT, the surface is often truncated for pelvis treatments due to the small field of view and thus changes in weight could go completely unnoticed without such metrics. This truncation is even more prevalent on Halcyon machines that are only equipped with the MV CBCT. When this technique is further developed to analyze the images as they are acquired instead of posttreatment, they could also prevent spine mistreatments from being aligned to the wrong vertebral body or errors in the executed couch translations after image guidance.

In clinical practice, more than one of these effects could be present at any given time, (e.g. patient weight loss and intrafraction patient motion). Additionally, in certain cases, one effect could even mask another, such as a slight decrease in the daily output of the beam cancelling out the effect from a decrease in patient thickness. We limited the present analysis to investigating one effect at a time because the purpose of this study was to demonstrate that large clinically significant changes in either factor result in a measurable response from the panel. Increases in a metric would then raise a flag for the covering physicist to investigate further. Future studies will look at defining reasonable thresholds when multiple changes are present.

We also measured both the MRD and *σ_RD_* from the exit images for six patients treated clinically. The main purpose of the patient analysis was to demonstrate the feasibility of our workflow and metrics on real patient data. From the results, we observed that both MRD and *σ_RD_* were sensitive to changes arising from gas bubbles and thus could be valuable metrics to include for clinical evaluations. In this study, we used a binary classification for the presence of gas and showed that these metrics showed significant changes with gas. Some overlap in the values occurred for images with and without gas likely because our binary classification meant that images with very small amounts of gas were characterized the same as those with large gas bubbles. A natural next step would be to measure the size of these bubbles and ascertain if the MRD and *σ_RD_* are correlated with increasing gas size. Retrospective replans could then be calculated using the deformed anatomy to determine at what threshold gas volume a significant dosimetric impact occurs. This would allow for the metrics measured from exit images to trigger an alert at the machine when a patient’s gas is beyond this threshold all without the therapists needing to examine the image or measure the gas volume themselves.

Interestingly, one patient did not have a significant difference in the values for either metric when gas was present. This may be due to an overall smaller amount of gas in any of their images as well as larger variations in their images without gas. This patient had a larger range of *σ_RD_* values when gas was not present than any of the other patients and had the largest MRD values for any of the images without gas. The cause of this inherent larger variation may be more apparent as we analyze larger patient sets.

**Figure 10:**
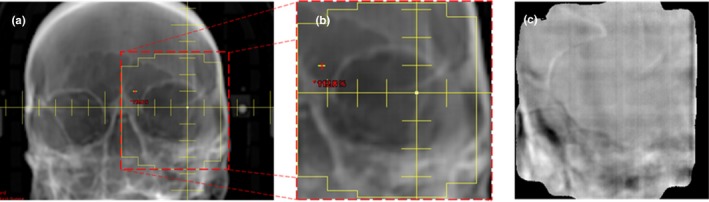
Digitally reconstructed radiograph (a), zoom‐in on the treated field (b), and relative difference (c) images are shown for one head and neck patient. The bones visible in the exit images could potentially be used to track intrafraction motion.

The qualitative examination of the patient’s anatomy from the exit dosimetry images clearly revealed the pubic symphysis. Minor changes in the symphysis positioning could be manually measured. While small shifts of 1‐2 mm are not critical for prostate patients, particularly since for these patients we align daily to the PTV and thus the relative position of nearby bony anatomy is expected to change, shifts of this magnitude could have a considerable clinical impact for head and neck patients or spine stereotactic body radiation therapy (SBRT) patients. To verify if anatomical structures could be qualitatively visualized in head and neck patients, the exit dosimetry images from the first treatment of an orbit patient treated on the Halcyon™ at our institution were also reviewed, see Fig. 10. The bones of the frontal skull and maxilla can be clearly seen in the exit images. These could potentially be used to track intrafraction patient motion to prevent treatment errors.

While we only examined the MRD and *σ_RD_* for six patients, the exit images from every patient treated at our institution on the Halcyon™ are automatically stored in ARIA and are available for analysis at any time. By examining the full set and normalizing each patient to their own specific baseline, it could be possible to note trends such as an increase in the beam output on a particular day. This would allow us to detect the instance of a change in beam output that occurred over the course of a day and thus in between daily QA checks. Examining a larger patient set will also allow us to better understand the causes of variation in these metrics and determine potential clinical thresholds.

This study had a few weaknesses, the primary of which was using the first fraction without noticeable amounts of gas as the baseline for the quantitative analysis of the patients’ images. Using an image predicted by the treatment planning system would be preferable since the baseline would be the actual patient position used for the plan. However Eclipse v15.1 cannot currently generate this predicted image. We planned to use the first fraction as the baseline, but for four of the six patients there were appreciable amounts of gas on day one and we did not want to penalize subsequent images for not having gas. We are working to develop a methodology to calculate the predicted image from the treatment plan within Eclipse or use a 3^rd^ party workflow to serve as the reference image for future studies and clinical implementation. Similar workflows have been developed by research groups and appear in the literature.^29^, ^38^

Additionally, in this current implementation, errors would not be detected until after a treatment fraction is delivered. For our initial implementation, we think this is adequate as it would serve to flag images for physics review and a deeper analysis rather than interrupting treatment for every anomaly. For certain errors such as weight loss, identifying trends over the course of several fractions would be enough for preventing treatment errors. In the long‐term, we plan to further develop this technique so that images can be analyzed as they are acquired such as at every specified number of control points allowing us to prevent potential mistreatments.

Another potential weakness of this study was the fact that our methodology assumed IMRT treatments with fixed fields. Previously at our institution the Halcyon™ was only being used to treat static field‐modulated plans, however, we recently expanded to using it for volumetric modulated arc therapy (VMAT) plans. In this situation, the anatomical landmarks we observed in the present analysis would not be visible in the integrated image because the gantry would move through treatment. To analyze transit images from VMAT plans, images would have to be generated and compared for the summation of the whole arc or extracted at a specified frequency^10^, ^11^ such as at each control point of a VMAT plan.^39^ This might not impact the evaluation of the quantitative metrics proposed in this study, however, the sensitivity of the panel to changes in output, patient weight, and patient alignment may differ if it is averaged over an entire arc.

Instead of the three metrics proposed in this study, gamma analysis could be used to evaluate the transit image accuracy. However, we selected not to include gamma analysis because of studies demonstrating its limited sensitivity to IMRT errors.^40^, ^41^, ^42^ We wanted a quick quantitative indicator that something has gone wrong which scales linearly with the size of the error. We believe the MRD and calculated shift accomplish this more efficiently than gamma analysis for output or buildup changes and lateral shifts, respectively.

In this study, we focused on finding the magnitude of change that was detectable. However these thresholds must be considered alongside of what magnitude of change is clinically relevant.^43^ The clinically relevant magnitude of change will vary with treatment site and technique and potentially even with each patient and as a result is more challenging to measure. Work by other groups have typically used gamma analysis with standard pass criteria (3%, 3 mm)^20^, ^25^, ^44^ to determine if measured transit images pass. One study using a 3%, 3 mm criteria had false positive alerts for 10 clinically irrelevant discrepancies,^19^ while in a separate clinical implementation, a gamma analysis with looser criteria of 4%, 4 mm for pixels above a 10% threshold was used but no patients in the trial ever had such large differences.^45^ Determining what limits should be used in our clinic to identify relevant errors will be an area of future study. One potential methodology for evaluating the clinically relevant magnitude of change for prostate patients would be to use a phantom with dynamic bladder and rectum compartments. A treatment plan could be generated using full bladder and empty rectum conditions, and then recalculated with varying levels of empty bladder and full rectum to determine at which point there is a significant dosimetric response. Then transit dosimetry images could be collected for the same set of bladder and rectum conditions to evaluate whether the panel detects significant differences.

In this analysis, we demonstrated the feasibility of extracting and analyzing exit dosimetry images from the Halcyon™ built‐in portal imager. To do this, we developed three quantitative metrics for easy comparison of daily images to baseline images, and then showed that these metrics were sensitive to small changes in beam output, patient position, and patient weight changes. In the next iteration of this study, we intend to develop a workflow for generating the exit dosimetry image prediction from Eclipse. This will produce a more reliable baseline for analyzing patient images daily and allow us to analyze the large volume of patient images we have already acquired. The long‐term goal of this study was to automate the analysis of these images in order to alert clinicians that the patient may require repositioning or replanning. In the final clinical implementation, our goal is to acquire these images for every patient on treatment and evaluate them in real‐time so that significant errors would trigger an immediate alert. This technique would be a valuable safety check through treatment as it would alert therapists if the patient moved during treatment, lost weight over the course of treatment, or if the beam output was different from the plan. These extra safety checks would be especially useful for the patient’s first fraction to ensure their initial setup and beam delivery match the intended plan and anatomical positioning. This could help mitigate potential problems where spinal patients are aligned to the wrong vertebrae or the MLC parameters are not correctly communicated to the machine.

## 5 CONCLUSIONS

Our results indicated that the imager was sensitive to large modifications in changes in output, patient weight, or patient alignment, and thus mid‐treatment changes all have the potential to be detected by the imaging panel. This study presents the first steps toward developing effortless image evaluation for all aspects of every patient’s treatment on the Varian Halcyon™.

## CONFLICTS OF INTEREST

Kevin L. Moore is the recipient of a Varian research grant, travel support, and Honoraria unrelated to this work.
